# Environmental surveillance of ESBL and carbapenemase-producing gram-negative bacteria in a Ghanaian Tertiary Hospital

**DOI:** 10.1186/s13756-022-01090-2

**Published:** 2022-03-16

**Authors:** Joseph Elikem Efui Acolatse, Edward A. R. Portal, Ian Boostrom, George Akafity, Mavis Puopelle Dakroah, Victoria J. Chalker, Kirsty Sands, Owen B. Spiller

**Affiliations:** 1 Cape Coast Teaching Hospital, Cape Coast, Ghana; 2 Medical Microbiology, Department of Infection and Immunity, School of Medicine, Cardiff University, Cardiff, UK; 3 Bacteriology Reference Department, UK Health Security Agency, London, UK; 4 Department of Zoology, Oxford University, Oxford, UK

**Keywords:** AMR, AFRICA, Ghana, Hospital environment, Acinetobacter, CRAB, ESBL, NDM, Carbapenemase, Infection prevention and control

## Abstract

**Background:**

The burden of antibiotic resistant infection is mainly felt in low-to-middle income countries, where the rate of antimicrobial resistance is largely under-surveyed and under huge pressure from unregulated, disparate and often self-guided access to antimicrobials. Nosocomial infections from hospital environments have been shown to be a particularly prevalent source of multi-drug resistant strains, yet surveillance of hospital environmental contamination is often not investigated.

**Methods:**

The study was prospective, observational and cross-sectional, sampling 231 high and low touch surfaces from 15th March to 13th April 2021, from five wards in the Cape Coast Teaching Hospital, Ghana. Microbial growth in the presence of vancomycin and either meropenem or cefotaxime was examined and bacterial species were identified by MALDI-TOF. The presence of common extended-spectrum β-lactamases (ESBL) and carbapenemase antimicrobial resistance genes (ARG) were identified through PCR screening, which were confirmed by phenotypic antimicrobial susceptibility determination. Isolates positive for carbapenem resistance genes were sequenced using a multi-platform approach.

**Results:**

We recovered microbial growth from 99% of swabs (n = 229/231) plated on agar in the absence of antimicrobials. Multiple sites were found to be colonised with resistant bacteria throughout the hospital setting. Bacteria with multi-drug resistance and ARG of concern were isolated from high and low touch points with evidence of strain dissemination throughout the environment. A total of 21 differing species of bacteria carrying ARG were isolated. The high prevalence of *Acinetobacter baumannii* carrying *bla*_NDM-1_ observed was further characterised by whole genome sequencing and phylogenetic analysis to determine the relationship between resistant strains found in different wards.

**Conclusion:**

Evidence of multiple clonal incursions of MDR bacteria of high sepsis risk were found in two separate wards for a regional hospital in Ghana. The prevalence of multiple *bla*_NDM_ carrying species in combination with combinations of ESBLs was particularly concerning and unexpected in Africa. We also identify strains carrying *tet*(X3), *bla*_VIM-5_ or *bla*_DIM-1_ showing a high diversity of carbapenamases present as a reservoir in a hospital setting. Findings of multi-drug resistant bacteria from multiple environmental sites throughout the hospital will inform future IPC practices and aid research prioritisation for AMR in Ghana.

**Supplementary Information:**

The online version contains supplementary material available at 10.1186/s13756-022-01090-2.

## Background

Antimicrobial resistance (AMR) is a recognized global health crisis with annual projections estimating up to 10 million lives being lost and costing $100 trillion globally by the year 2050 [1]. Of particular concern is antibiotic resistance among Gram-negative bacterial species which presents an ever-increasing threat due to the diverse pathologies they are capable of causing, as well as their acquired and intrinsic resistance mechanisms which render many existing antibiotics ineffective. This leads to increased morbidity, mortality, and adverse patient outcomes [2]. In light of this problem, the World Health Organization (WHO) published the global Pathogen Priority List which contains selected bacteria pathogens for which new treatments are urgently needed. The ESKAPE (*Enterococcus faecium, Staphylococcus aureus, Klebsiella pneumoniae, Acinetobacter baumannii, Pseudomonas aeruginosa,* and *Enterobacter* spp.) pathogens, out of which four are Gram-negative organisms designated as “critical priority status” [3] further highlights their importance in the spread and propagation of AMR and disease burden. These pathogens have been well documented for their significant contribution to mortality in a wide range of clinical presentations such as ventilator-acquired pneumonia, central line-associated bacteremia [4] nosocomial meningitis [5], neonatal sepsis [6], and bloodstream infections [7, 8], with most of these being hospital-associated infections [9]. Additionally, several strains of these bacterial species have propagated worldwide with the concomitant spread of the mobile resistance genes they harbour, especially those conferring resistance to key classes of antibiotics namely the β-lactam class (including carbapenems) of antibiotics [10–16]. Carbapenem antibiotics within this class are more expensive and mostly reserved as second or last-line options for hard-to-treat infections or when first-line therapy proves to be ineffective in Ghana [17]. The WHO AWaRe (Access, Watch, and Reserve) classification categorizes most third-generation cephalosporins and carbapenems as “Watch” drugs based on their likely potential to induce antibiotic resistance which could have negative consequences for wider populations [18]. Extended-Spectrum β-Lactamase (ESBL) and carbapenemase genes are implicit in this occurrence and are known to spread relatively rapidly within and between countries and continents [6, 19].

In Africa, data on the prevalence and characterization of healthcare-associated infections (HAI’s) caused by these organisms is available though not comprehensive [20–22]. Additionally, where infectious diseases have a high burden coupled with sometimes inadequate laboratory capacity, the effects of AMR may be higher especially in low- and low-middle-income countries in the region [23]. Other factors such as high poverty rates, burdened healthcare systems, and poor infection prevention and control practices (IPC) may further exacerbate the issue [24]. In Ghana specifically, several studies exist on phenotypic and molecular characterization of these priority pathogens mainly with a focus on clinical isolates. Intermediate to high rates of resistance to cephalosporins and carbapenems similar to estimates in the continent and globally were reported in these studies despite limitations of small sample sizes in some cases [25–33]. With regards to surveillance of AMR from hospital environments, research studies exist [34–37] but are inadequate especially for Gram-negative pathogens considering the important role hospital environments can play in transmission. The presence of resistant bacteria in hospital environments are a critical component of nosocomial infection. Contaminated intermediate objects represent a common segue of transmission between patients, from visitors to patients, or from healthcare workers to patients [38, 39], which also impact on the choice of antibiotic prophylaxis for surgeries [40]. The Ghanaian National policy on IPC overseen by the Ministry of Health delineates roles and good practices to be followed among all levels of healthcare [38] and emphasizes the importance of environmental cleaning and disinfection and generation of robust data on monitoring and evaluating these practices to advise the MOH research agenda on priority areas of IPC at either national, regional, and district levels.

This study investigated the presence and prevalence of ESBL- and carbapenemase-carrying bacterial species by swabbing "high touch" and “low touch” areas in five wards in the Cape Coast Teaching Hospital, Ghana. We performed microbial culture and PCR on the total growth to detect the presence of ESBL genes (*bla*_CTX-M-15_, *bla*_OXA-1_, *bla*_TEM_, *bla*_SHV_) and carbapenemase genes (*bla*_NDM_, *bla*_OXA-48_-like, *bla*_KPC_). Positive samples were characterised by MALDI-TOF to identify the bacterial isolate containing the antimicrobial resistance genes (ARG), and isolates containing carbapenemase genes were further analysed by whole- genome sequencing (WGS) and antibiotic susceptibility testing (AST) with a range of antibiotics.

## Methods

### Study design

This study aimed to establish environmental antimicrobial resistance surveillance in the Cape Coast Teaching Hospital (CCTH) in Ghana to enable adequate identification of resistance patterns and genetic mechanisms of bacterial isolates recovered from fomites classified as “high touch” and “low touch” areas in inpatient wards and to determine their potential as sources of these pathogens and implications in hospital-acquired infections. The study was a prospective, observational, cross-sectional study that employed sampling from 15 March to 13 April 2021. A total of 231 swabs were collected from five wards in CCTH namely, Obstetrics and gynaecology (OG), Neonatal Intensive Care Unit (NICU), Female Medical Ward (FMW), Accident and Emergency Ward (AE), and the Paediatric Ward (PW). These were selected purposively based on local clinical susceptibility data (based on CLSI M-100 30ed. Guidelines) [41] indicating a high occurrence of potential ESBLs i.e., resistance to third-generation cephalosporins (cefotaxime, ceftriaxone, or cefuroxime) for the year 2020 via the WHONET software (unpublished data).

CCTH is a major tertiary hospital located in the Central Region of Ghana, West Africa. It is a 400-bed capacity institution that offers specialist services and serves a population of 170,000 in the Cape Coast Metropolitan area [42]. It also receives referrals from within the Central, Western and other regions in the country.

Swab sites were selected based on guidance from the National IPC policy and published literature. Items were further classified as “high touch” and “low touch” areas based on how likely healthcare workers and patients would encounter them based on the investigators’ judgments and observations, including:

1. *Low touch areas* IV stands, bed wheels, bedpans or trays, sphygmomanometer cuffs, desk surfaces, waiting area chair handles, thermometers and stethoscopes.
2. *High touch areas* Bed handles, mattress, pillows, bedside carts, bedside cabinet tops and drawers, computer keyboards, window levers, light switches, wall socket switches, tap handles, and washroom door handles.

Items were swabbed between 9:00 am and 2:00 pm on weekdays. Ward staff were not informed of the swabbing date to capture the true prevalence of gram-negative bacteria (GNB) after routine ward cleaning. Swabs were stored in Amies charcoal transport medium and were refrigerated at 4 °C, shipped via a courier service per UN3373 regulations to Cardiff University and stored at 4 °C prior to testing.

### Microbial culture and PCR of target antibiotic resistance genes

Swabs were streaked onto three chromogenic agars; no antibiotics, vancomycin and cefotaxime (VC, 10 mg/L and 1 mg/L) to select for cefotaxime resistance an indicator of ESBLs, and vancomycin and meropenem (VM, 10 mg/L and 1 mg/L) to screen for the presence of carbapenemases. Agar plates were incubated aerobically for 48 h at 37 °C.

VC and VM plates recording growth were streaked and 10 µl from the first quadrant (mixed microbial community) was suspended into 150 µl of sterile distilled molecular grade water. Three PCR Master Mix preparations were made: ESBL Multiplex Master mix was used in detecting *bla*_TEM_* bla*_*SHV*_*, bla*_OXA-1_ [43], Carbapenemase Multiplex Master mix for detecting *bla*_NDM_, *bla*_OXA-48-__like_ variants, and *bla*_KPC_ and CTX-M Master mix for detection of *bla*_CTX-M-15_ (see Additional file 1: Table S1 for primer sequences and Additional file 1: Table S2 for conditions).

One microlitre of the bacterial suspension was added to 24 µl of each of the three PCR Mastermixes, PCR and gel electrophoresis (300 V for 40 min) were performed on isolates. Those yielding positive for any of the tested resistance genes were further sub-cultured to obtain pure single colonies which were then re-screened to confirm the presence of the gene of interest.

Positive isolates were identified using a Microflex LT MALDI-TOF MS (Bruker Daltonik) with α-cyano-4-hydroxycinnamic acid matrix (Sigma–Aldrich). Bacterial isolates were stored in TS/72 beads (Technical Service Consultants) at − 80 °C and the original swabs were stored at 4 °C.

Descriptive statistics were used in determining the prevalence of ESBL- and carbapenemase-producing GNB recovered from the ward environments as well the number and types of resistance genes they harboured. Microbial growth and PCR data were recorded and analysed using Microsoft Excel.

### Antimicrobial susceptibility testing (AST)

Minimum inhibitory concentrations (MIC) were determined by agar dilution with a multipoint inoculator (MAST Uri-dot) using cation adjusted Mueller–Hinton agar for meropenem, ceftazidime, tigecycline, gentamicin and colistin. *Escherichia coli* ATCC 25922, *Klebsiella pneumoniae* ATCC 700603, *Pseudomonas aeruginosa* ATCC 2785, and *Escherichia coli* ATCC 13846 (*mcr1* positive) were used for quality control and the results were interpreted according to the EUCAST v11 guidelines and ECOFF values where applicable [44]. Agar dilution was used for colistin MIC as concordance between agar and microbroth dilution methods has previously been published [45].

### Whole-genome sequencing (WGS)

Isolates with a carbapenemase gene were selected for WGS using an Illumina MiSeq as described in Sands et al. [6]. Isolates were selected for additional long read sequencing guided by the initial short read bioinformatics analysis. gDNA was prepared as previously described [6] using the Qiagen QIAamp DNA kit and extracted on the QIAcube platform (Qiagen). gDNA was quantified using the dsDNA HS assay kit and the Qubit 4.0 fluorometer (Thermofisher), purified and concentrated using SPRI beads (Mag-Bind TotalPure, Omega) at a 1:1 ratio with a final elution volume of 15 µl to achieve an optimal range between 40 and 60 ng/µl. gDNA was re-quantified with the dsDNA BR assay kit. Genomic libraries were prepared using the Rapid Barcoding Kit (SQK-RBK004; Oxford Nanopore), sequenced on a R9.4 flow cell using a MinION (Oxford Nanopore Technologies) and basecalling was performed using Guppy v4.0.9 within MinKNOW v20.06.4.

### Short read bioinformatics analysis

Fastq reads were subject to quality control (QC) analysis and trimming using fastqc (v0.8.11) [46] and trimgalore (v0.5.0) [47] respectively. Reads were assembled into contigs using shovill (v0.9.0) [48] and assembly metrics were assessed using quast (v5.0.2) [49]. ABRicate (v0.9.7) [50] (> 98% coverage and identity) was used to detect ARG and virulence factors (VF) with accompanying databases Resfinder and VFDB. In silico MLST was determined using *mlst* (v2.17.6) [51] and the Pasteur scheme was used [52]. Genomes (contigs) were annotated using Prokka (v1.14.0) [53] and the resulting.gff files were used to create a core genome alignment of *Acinetobacter* spp. using Roary (v3.12.0) [54], and IQ-tree (v2.0) [55] was used to create a maximum likelihood phylogeny. ModelFinder within IQ-tree utilised the GTR+F+I+G4 model and 1000 bootstrap replicates were performed. For SNP analysis a local reference genome was selected for additional long read sequencing using Oxford Nanopore MinION (details described below) to produce a high-quality genome. Snippy (v4.4.5) [56] was used to map the fastq reads against the reference. The SNPs were aligned using snippy-core and the SNP sites were extracted using snp-sites (v2.5.1) [57]. Recombination events were removed with Gubbins (v.2.3.4) [58] and IQ-tree (v2.0) [55] was used to create a maximum likelihood phylogeny. Pairwise SNP distances were generated using snp-dists (v0.7) [59]. Phylogenetic trees were mid-rooted, visualised and annotated using iTOL (v5.7) [60].

### Long read bioinformatics analysis

Long reads were demultiplexed using porechop (v0.2.4) [61] and assembled against corresponding short reads generated from the Illumina MiSeq using Unicycler (v0.4.7) [62] with default parameters. The hybrid assembly was assessed using quast (v5.0.2) and ABRicate (v0.9.7) [50] (> 98% coverage and identity) was used to detect antimicrobial resistance genes (ARG). The contig containing the ARG of interest was exported to a separate fasta file for comparative analysis using Bandage (v0.8.1) [63]. Plasmid sequences were uploaded to PLSDB [64] and mash dist search strategy was used to assess for similar plasmids in the database. The MobileElementFinder database (v1.0.2) was downloaded and ABRicate [50] was used to search for the mobile genetic elements (MGE) genomic context of ARG.

### Data for patient isolates

Comparison for data collected between 1st January and 30th June 2021, for bacterial isolates submitted to the local microbiological diagnostic laboratory at CCTH were examined for comparison to the in-depth swab analysis performed at Cardiff University. Ethical approval to include patient isolate data in the analysis was granted (CCTHERC/EC/2021/027) by the local ethical committee at CCTH. Data was restricted to ward origin of isolates, bacterial species and AMR investigation. All patient isolates included were inoculated on MacConkey agar with urine isolates inoculated exclusively on Cysteine Lactose Electrolyte Deficient (CLED) agar (Himedia, India) plates for 24 h at 35 ± 2 °C. Sub-culturing was employed to obtain pure colonies which were subjected to gram-staining and microscopy. Biochemical analysis was performed under aerobic conditions and these plates were incubated for 24 h at 35 ± 2 °C and included metabolism of indole, citrate, urea, glucose, lactose, hydrogen sulphide production, gas, oxidase presence and motility (differential determination given in Additional file 1: Table S3). Antibiotic susceptibility testing (AST) via the disk diffusion method using a 0.5 McFarland standard bacterial suspension of pure isolates on Muller Hinton agar (Himedia, India) incubated for 24 h aerobically at 35 ± 2 °C. AST data were collated by WHONET software (version 21.11.24) utilising CLSI 2021 breakpoints, and the following parameters: 1. analysis type – study = RIS and test measurements, antibiotics = CRO, CTX, SAM, TZP; 2. Organisms – ALL = All organisms; 3.All other parameters were used in their default.

## Results

### Microbial culture and PCR of environmental swabs

The largest number of swabs collected was in the PW (n = 71) followed by the NICU (n = 53). For the FMW, OG and AE n = 36, 38, and 33 swabs were collected respectively (Table 1). We recovered bacterial growth from all but 2 swabs (n = 229/231) collected during this study when plated onto non-selective agar, in the absence of antibiotics. Surfaces from OG produced the highest level of GNB growth on both VC and VM agar (Table 1). Swabs taken from the FMW produced the least GNB growth on VC agar (n = 24/36, 67%) and second to least growth on VM (n = 17/36, 47%). Surfaces from NICU produced the least growth on VM agar (n = 23/53, 43%) (Table 1).

**Table 1:** The number of swabs collected per ward and the number/percentage of swabs producing bacterial growth on agar supplemented with vancomycin and cefotaxime (VC) and/or agar supplemented with vancomycin and meropenem (VM)

Ward	Number of swabs taken	Number recording growth VC	Number recording growth VM	% Growth VC	% Growth VM
Female medical ward (FMW)	36	24	17	67	47
Obstetrics and gynaecology ward (OG)	38	36	31	95	82
Paediatric ward (PW)	71	61	53	86	75
Neonatal ICU ward (NICU)	53	32	23	60	43
Accident and emergency ward (AE)	33	27	17	82	52
Total	231	180	141	78%	61%

Swabs (n = 231) were collected from a total of 28 different surfaces (Additional file 1: Table S4) with one-third (n = 77/231, 33%) collected from the immediate surroundings of patient beds (sheets, pillows, bed wheels, bed pans, etc., Additional file 1: Table S4). The majority of swabs collected around patient beds produced growth on both agars supplemented with antibiotics (VC n = 71/77, 92% and VM n = 61/77, 86%). Computer keyboards and desk surfaces produced very high GNB growth rates on VC agar (n = 12/13, 92% and n = 11/13, 85% respectively), similar to tap handles and thermometers (n = 14/17, 82% and n = 9/10, 90%). Surface swabs across computer keyboards, desks, tap handles and thermometers produced a similar GNB growth recovery on VM (between 60 and 71%, Additional file 1: Table 4).

**Fig. 1:**
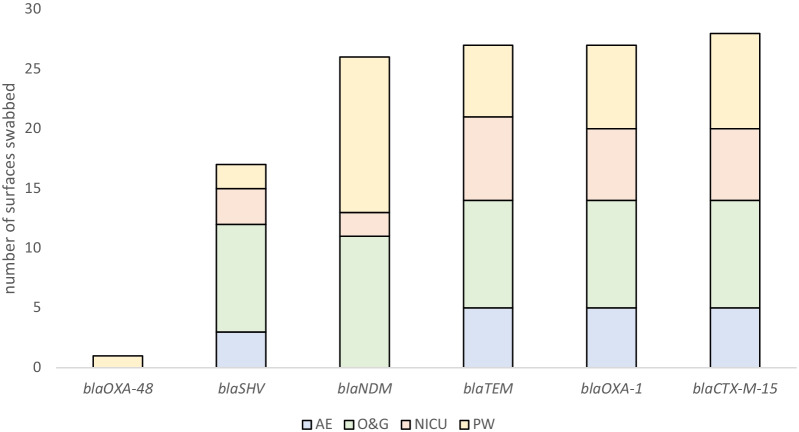
A stacked bar graph coloured per ward showing the number of hospital surface swabs PCR positive for each antibiotic resistance gene screened. The total number of swabs processed was 231

In total, 26 surfaces (n = 26/231, 11%) were positive for *bla*_NDM_ (n = 13/26 50% PW, n = 11/26 42% OG and n = 2/26 8% NICU), 12/26 were collected from the immediate surroundings of patient beds, two from bed wheels (NICU and PW), three from computer keyboards and desks from different wards (OG and PW), three from tap handles or washroom doors from different wards (OG and PW), as well as assorted other sites including a thermometer (PW), sphygmomanometer (OG), medicine trolley (NICU) IV stand (PW) and a wall socket (OG) (Fig. 1). One surface (PW-0012) was positive for multiple ARG including *bla*_NDM_, *bla*_OXA-48_-like (the only surface positive for *bla*_OXA-48-_like), *bla*_SHV_ and *bla*_CTX-M-15_. There were 28 surfaces (n = 28/231, 12%) positive for at least one ESBL gene (Fig. 1) and five surfaces (n = 3 OG, n = 2 PW) contained both carbapenemase and ESBL ARG genes.

### Gram-negative bacterial (GNB) isolates positive for ESBL and/or carbapenemase ARG

**Fig. 2:**
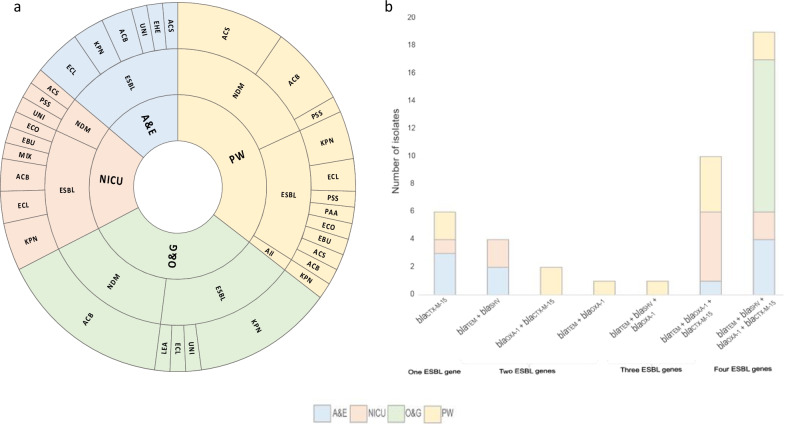
**a** Sunburst diagram summarising bacterial species identified (outer ring); ACB, *Acinetobacter baumannii complex*; ACS, *Acinetobacter* spp.; ECO, *E. coli*; EBU, *Enterobacter bugandensis*; ECL, *Enterobacter cloacae*; EHE, *Escherichia hermannii*; KPN, *Klebsiella pneumoniae*; LEA, *Leclercia adecarboxylata*; MIX, *Mixta calida*; PAA, *Pantoea agglomerans*; PSF, *Pseudomonas fulva*; PSS, *Pseudomonas stutzeri*; UNI, Unidentified. The carriage of ESBL and/or carbapenemase genes (middle ring); ESBL (*bla*_CTX-M-15_ and/or *bla*_OXA-1_ and/or *bla*_SHV_ and/or *bla*_TEM_), All (all ESBL genes identified and *bla*_NDM_ and *bla*_OXA-48_-like). The ward the sample was collected from (inner ring); AE, accident and emergency; OG, obstetrics and gynaecology; NICU, Neonatal Intensive Care Unit; PW, paediatric ward

A total of 71 bacterial isolates from 21 bacterial species (including three isolates into the ‘unidentified’ category where the MALDI-TOF failed to accurately speciate the colony) containing at least one target ESBL (n = 43, 61%) and/or carbapenemase (n = 28, 39%) gene (Table 2) were recovered. Ten isolates from six GNB species were recovered from the AE, all of which contained at least one ESBL gene (Fig. 2a), as were 13 GNB isolates from NICU, 10 containing ESBL genes, and three with *bla*_NDM_ genes (Fig. 2a). In OG 8/11 ESBL containing isolates were *K. pneumoniae* whereas *A. baumannii* (n = 11/11, 100%) isolates carried *bla*_NDM_ (Fig. 2a). The highest frequency of isolates recovered was from PW (n = 24), with a similar split between GNB isolates carrying ESBL genes and carbapenemase genes. Eight different species were identified harbouring ESBL in PW and similarly to OG the most frequent species was *K. pneumoniae* (n = 3/11, 27%) (Fig. 2a)*.*

**Table 2:** The number of Gram-negative bacterial isolates identified, separately according to the number of ESBL positive isolates and/or *bla*_NDM_/*bla*OXA-48-like positive

Species	Total n =	*bla*_NDM_/*bla*_OXA-48_	ESBL
*Acinetobacter baumannii*	18	14	4
*Acinetobacter baylyi*	2	0	2
*Acinetobacter indicus*	3	3	0
*Acinetobacter johnsonii*	1	1	0
*Acinetobacter junii*	1	1	0
*Acinetobacter nosocomialis*	3	3	0
*Acinetobacter pittii*	1	0	1
*Acinetobacter spp*	1	1	0
*Acinetobacterr bereziniae*	1	1	0
*Acinetobacterr towneri*	1	1	0
*Escherichia coli*	2	0	2
*Enterobacter bugandensis*	2	0	2
*Enterobacter cloacae*	8	0	8
*Escherichia hermannii*	1	0	1
*Klebsiella pneumoniae*	17	1	17
*Leclercia adecarboxylata*	1	0	1
*Mixta calida*	1	0	1
*Pantoea agglomerans*	1	0	1
*Pseudomonas fluva*	1	0	1
*Pseudomonas stutzeri*	2	0	2
Unidentified	3	1	2

ESBL indicates at least one of*, bla*_CTX-M-15_, *bla*_TEM_, *bla*_SHV_, *bla*_OXA-1_

When delineating the occurrence of ESBL genes, six isolates from five different species carried *bla*_CTX-M-15_ only (Fig. 2b). Three different groups of isolates were detected containing dual ESBL genes (*bla*_TEM_ + *bla*_SHV_, *bla*_OXA-1_ + *bla*_CTX-M-15_, *bla*_TEM_ + *bla*_OXA-1_) from AE, NICU, and OG, however the majority of isolates (n = 30/43, 70%) contained ≥ 3 ESBL genes, and n = 19 isolates carried all four target ESBL ARG (Fig. 2b), including all 14 K*. pneumoniae* (10 from OG, 2 from PW, 2 from NICU), three *Enterobacter cloacae* (2 from AE, 1 from OG)*,* and two *A. baumannii* complex (both from AE: one *A. baumannii* and one *Acinetobacter pittii*). Most carbapenemase genes encountered in the study were detected from *Acinetobacter* isolates (n = 25/28), the remaining belonging to *Pseudomonas stutzeri* (n = 2/28) and *Klebsiella pneumoniae* (n = 1/28). The carbapenemase-carrying *K. pneumoniae* was also the only isolate positive for *bla*_OXA-48_-like, and the only isolate carrying all five target ARG selected for the study. Due to the multi-drug resistance (MDR) focus of this study the carbapenemase isolates were selected for further characterisation by AST and WGS.

### Species diversity, sequence type (ST) analysis, and genomic isolate relatedness

**Fig. 3:**
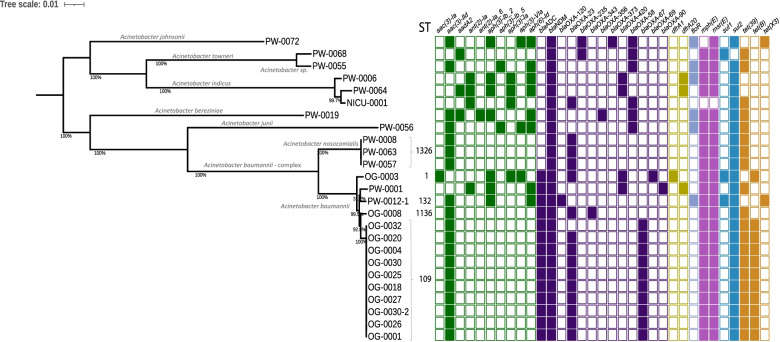
A mid-point rooted core genome phylogenetic tree of *bla*_NDM_ *positive Acinetobacter* isolates. Leaf labels are isolate codes with the ward abbreviation (PW, paediatric ward; OG, obstetrics and gynaecology; NICU, neonatal intensive care) and swab number. The sequence type (ST) follows the leaf node where applicable. The presence/absence of antibiotic resistance genes is colour coded per class; aminoglycoside (green), β-lactam (purple), trimethroprim (olive), phenicol (lilac), macrolide (pink), sulphonamide (blue), tetracycline (orange)

There were 28 isolates with WGS data, and n = 25 isolates were *Acinetobacter,* from eight different species. Raw sequencing reads from NICU-0042 (unidentified on the MALDI-TOF) were excluded from analysis after failing QC. The most frequent species identified was *A. baumannii* (n = 14) (Table 3, Fig. 3)*,* three isolates were identified as *Acinetobacter indicus* and three were identified as *Acinetobacter nosocomialis.* A single isolate each represented five other *Acinetobacter* species (Table 3). Two isolates were identified as *Pseudomonas stutzeri*, and although both were negative for *bla*_NDM-1_ by WGS analysis, one was found to contain *bla*_VIM-5_ and the other was found to carry *bla*_DIM-1_ and were therefore kept within the dataset for analysis. Additionally, one carbapenemase positive isolate was identified as *K. pneumoniae* (ST152) and contained both *bla*_NDM-1_ and *bla*_OXA-48_-like.

**Table 3:** A summary of each isolate with whole genome sequencing (WGS) data

Isolate code, location in ward and bacterial species Identification (ID)			Antibiotic phenotypic data MIC, ug/ml					Carbapenemase ARG			Number of antibiotic resistance genes per class										Virulence factors	
Isolate code	Location	ID	MER	COL	CEF	TIG	GEN	NDM	OXA-48	Other CARB ARG	AMG	BLA	RIF	TRI	PHE	FOS	MAC	FLU	SUL	TET	VF	BAVF
NICU0001	Cot handle	ACI	32	0.125	128	0.5	16	Y	N	*bla*OXA-23*, bla*OXA-67	2	3	0	0	0	0	0	0	1	1	ND	ND
NICU0008	Bed wheels	PSS	64	1	128	1	0.5	N	N	*bla*DIM	0	0	1	0	0	0	0	0	0	0	ND	ND
OG0001	Bed handle	ACB	256	1	> 256	0.5	256	Y	N	*bla*OXA-23*, bla*OXA-67	1	4	0	0	0	0	2	0	1	2	37	5
OG0003	Mattress	ACB	256	1	> 256	2	256	Y	N	*bla*OXA-23	4	5	0	1	0	0	2	0	2	1	36	4
OG0004	Pillow	ACB	128	1	> 256	0.5	128	Y	N	*bla*OXA-23*, bla*OXA-67	1	4	0	0	0	0	2	0	1	2	32	5
OG0008	Bedside cabinet	ACB	64	1	> 256	2	128	Y	N	*bla*OXA-23*, bla*OXA-699	1	4	0	0	0	0	2	0	1	1	33	4
OG0018	Sphygmomanometer	ACB	256	1	> 256	0.5	256	Y	N	*bla*OXA-23*, bla*OXA-67	1	4	0	0	0	0	2	0	1	2	35	5
OG0020	Desk surface	ACB	256	1	> 256	0.5	256	Y	N	*bla*OXA-23*, bla*OXA-67	1	4	0	0	0	0	2	0	1	2	34	5
OG0025	Desk surface	ACB	128	1	> 256	0.5	256	Y	N	*bla*OXA-23*, bla*OXA-67	1	4	0	0	0	0	2	0	1	2	33	5
OG0026	Washroom door handle	ACB	256	1	> 256	1	256	Y	N	*bla*OXA-23*, bla*OXA-67	1	4	0	0	0	0	2	0	1	2	35	5
OG0027	Tap handle	ACB	256	1	> 256	1	256	Y	N	*bla*OXA-23*, bla*OXA-67	1	4	0	0	0	0	2	0	1	2	37	5
OG0030	Bedsheet	ACB	128	1	> 256	0.5	256	Y	N	*bla*OXA-23*, bla*OXA-67	1	4	0	0	0	0	2	0	1	2	33	5
OG0030.2	Bedsheet	ACB	256	1	> 256	0.5	128	Y	N	*bla*OXA-23*, bla*OXA-67	1	4	0	0	0	0	2	0	1	2	33	5
OG0032	Wall socket switch	ACB	256	1	> 256	0.5	> 256	Y	N	*bla*OXA-67	1	3	0	0	0	0	2	0	1	2	31	5
PW0001	Bed handle	ACB	128	1	> 256	1	128	Y	N	*bla*OXA-90	3	4	0	1	0	0	2	0	1	1	33	4
PW0006	Mattress	ACI	64	0.06	256	0.5	16	Y	N	blaOXA-420	4	2	0	1	1	0	2	0	1	0	ND	ND
PW0007	Pillow	PSS	ND	ND	ND	ND	ND	N	N	*bla*VIM-5	1	3	0	1	0	0	2	1	0	0	ND	ND
PW0008	Pulse oximeter	ACN	ND	ND	ND	ND	ND	Y	N	*bla*OXA-23	1	2	0	0	0	0	2	0	1	1	6	2
PW0012.1	Bedside cabinet	ACB	64	0.5	> 256	8	256	Y	N	ND	3	4	1	0	1	0	2	0	2	1	31	3
PW0012.2	Bedside cabinet	KPN	128	32	128	2	64	Y	Y	ND	3	5	0	2	0	1	2	3	2	1	49	0
PW0019	Tap handle	ACZ	256	2	> 256	4	> 256	Y	N	*bla*OXA-356	5	4	0	0	1	0	2	0	1	2	ND	ND
PW0055	IV stand	ACT	64	0.5	> 256	0.5	128	Y	N	*bla*OXA-58	3	2	0	0	1	0	2	0	1	1	ND	ND
PW0056	Bed sheet	ACJ	64	8	> 256	1	> 256	Y	N	*bla*OXA-58	4	2	0	0	1	0	2	0	1	1	ND	ND
PW0057	Thermometer	ACN	128	0.5	> 256	0.5	128	Y	N	*bla*OXA-23	1	2	0	0	0	0	2	0	1	1	6	2
PW0063	Computer keyboard	ACN	128	1	> 256	0.5	128	Y	N	*bla*OXA-23	1	2	0	0	0	0	2	0	1	1	5	2
PW0064	Washroom door handle	ACI	32	0.125	256	0.25	8	Y	N	*bla*OXA-420	5	2	0	1	0	0	2	0	1	0	ND	ND
PW0068	Bed wheels	ACS	32	0.125	> 256	0.25	0.5	Y	N	*bla*OXA-235, *bla*OXA-58	1	3	1	0	0	0	2	0	1	1	ND	ND
PW0072	Pillow	ACO	16	0.125	256	0.5	64	Y	N	*bla*OXA-58, *bla*OXA651	4	4	1	0	1	0	1	0	1	1	ND	ND

The isolate code, location species, carbapebemase ARG, antibiotic phenotypic data, number of ARG per antibiotic class, total virulence factor genes (VF) and biofilm associated virulence factors (BAVF) are listed

^*^Species: ACI, *Acinetobacter indicus*; PSS, *Pseudomonas stutzeri*; ACB, *Acinetobacter baumannii*; ACN, *Acinetobacter nosocomialis*; KPN, *Klebsiella pneumoniae*; ACZ, *Acinetobacter berenziniae*; ACT, *Acinetobacter towneri*; ACJ, *Acinetobacter junii*; ACS, *Acinetobacter* sp.; ACO, *Acinetobacter johnsonii*

Antibiotic phenotypic data: MER, meropenem; COL, colistin; CEF, cefotaxime; TIG, tigecycline; GEN, gentamicin

Antibiotic resistance genes: AMG, aminoglycoside; BLA, β-lactam; RIF, rifampicin; TRI, trimethoprim; PHE, phenicol; FOS, fosfomycin; MAC, macrolide; FLU, fluoroquinolone; SUL, sulphonamide; TET, tetracycline

**Fig. 4:**
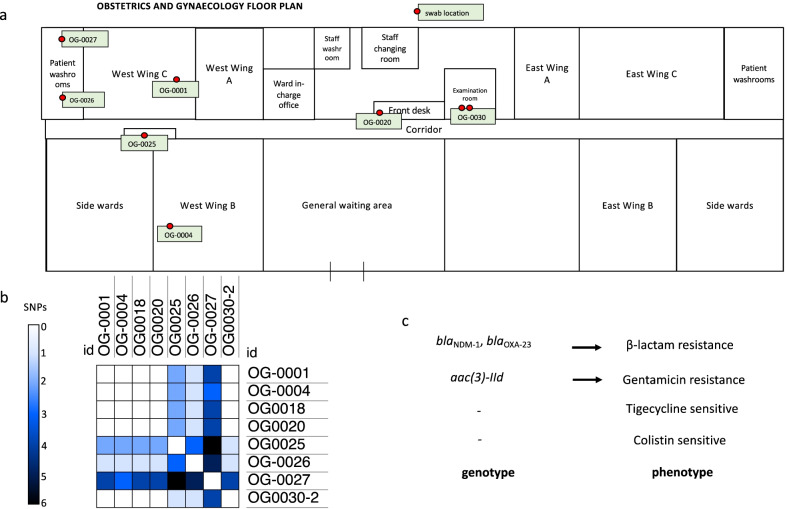
**a** An outline of the obstetrics and gynaecology ward with red dots indicating the locations of the swabs containing the cluster of ST109 *A. baumannii.* **b** A SNP heatmap revealing the pairwise SNPs between the isolates with sufficient genome coverage for analysis. **c** A phenotype to genotype linkage for the antibiotics tested, where β-lactam resistance includes antibiotics meropenem and cefotaxime

The most frequent ST for *A. baumannii* was ST109 (ST542 in the Oxford scheme) with n = 10 isolates (Fig. 3). All ten ST109 *A. baumannii* isolates were isolated from hospital swabs collected on the same day from the same ward, OG (Fig. 4a). Of the eight isolates of sufficient genome coverage for SNP analysis, the pairwise SNP distance was ≤ 6 SNPs. The most genetically distant ST109 isolate, OG-0027, was isolated from a tap handle in a patient washroom and was between 3 and 6 pairwise SNPs distant from the remaining ST109 isolates. Four isolates had 0 pairwise SNPs (OG-0001, OG-0004, OG-0018, and OG-0020) and these were isolated from samples collected from both low touch and high touch surfaces including patient bed materials across the ward from west wing B to west wing C, a sphygmomanometer and a desk surface used by staff at the general waiting area suggesting a transmission cluster and spread of carbapenem resistant *A. baumannii* (CRAB) (Fig. 4ab). Each ST109 isolate was resistant to β-lactam antibiotics tested (meropenem and cefotaxime) and gentamicin, whereas all were sensitive to colistin and tigecycline (Table 3, Fig. 4c).

In silico virulence factors (VF) were screened for in *A. baumannii* isolates and we were specifically screening for the presence of VF associated with biofilm production including biofilm-associated protein (*bap*) gene, *ompA, csuE, fimH, epsA, bfmS, ptk, pgaB, csgA, kpsMII* and *bla*PER-1 (a β-lactamase shown to enhance biofilm formation and attachment, especially to epithelial cells). All ST109 *A. baumannii* contained the greatest number of biofilm-related genes (*bap, bfmS, csuE, ompA* and *pgaB*), whereas the four single ST *A. baumannii* isolates (ST1, ST78, ST132 and ST1136) did not carry *bap.* The only other notable identification of VFs from in silico screening of Acinetobacter spp, was the identification of both *bfmS* and *ompA* in all three *A. nosocomialis* strains isolated from PW (Table 3)*.*

Other in silico STs detected for *Acinetobacter* included four *A. baumannii* isolates each of a different ST; ST1, a globally disseminated strain clonal complex (CC) 1 from OG, ST78 and ST1136 also from OG, and ST132 from PW. All isolates (n = 3) identified as *Acinetobacter nosocomialis* were typed as ST1326 and were from the PW. There were two sampling time points whereby the first *A. nosocomialis* isolate was recovered from swabbing the insides surfaces of the finger clip of a pulse oximeter in the west wing of the ward*.* One week later two additional *A. nosocomialis* isolates were recovered from a thermometer tip and handle in the west wing and a keyboard and mouse at the general nursing station. Although a small sample size, pairwise SNP distances suggest that these isolates are within 10 SNPs and are therefore likely to be related in a transmission cluster given the locality within the same ward and time frame. For this analysis the internal short read reference genome was utilised (PW0008) as whilst scanning the NCBI database and the PubMLST database [72] for an appropriate reference genome for SNP calling, we found only one ST1326 *A. nosocomialis* genome from a neonatal blood culture collected in 2017 in Nigeria [6] however this was approximately 10,000 SNPs distant from the PW ST1326 *A. nosocomialis.*

### Mechanisms of antimicrobial resistance in *bla*_NDM-1_ positive isolates

We performed AST via determination of the minimum inhibitory concentrations (MIC) for all isolates subject to WGS analysis. Due to the selective screening of ARG during this study and the focus for the detection of MDR bacteria, we tested meropenem, gentamicin, cefotaxime, colistin and tigecycline. All isolates were resistant to meropenem with the MIC range 16–256 mg/L (Table 3) (n = 27, 100%, it was not possible to test one *A. nosocomialis* and one *P. stuzteri* isolates due to recovery issues from −80 °C).

The *K. pneumoniae* isolate characterised by WGS and MIC (PW0012.2) contained *bla*_CTX-M-15_, *bla*_SHV-1,_ *bla*_OXA-1_ in addition to *bla*_NDM-1_ and *bla*_OXA-48_-like, was resistant to meropenem (MIC = 128 mg/L), cefotaxime (MIC =  > 256 mg/L), gentamicin (MIC = 64 mg/L) and colistin (MIC = 32 mg/L) although *mcr* genes were not detected nor were mutations in the *mgrB* gene often conferring resistance to colistin in *K. pneumoniae*, this isolate was susceptible to tigecycline (MIC = 2 mg/L) (Table 3). Hybrid de novo genome assembly (short read and long read sequences) for PW0012.2 revealed the *bla*_NDM-1_ and *bla*_OXA-48_ were located on the same 273,549 bp IncHI1B/IncFIB plasmid along with six other ARG from four antibiotic classes (Table 4).

**Table 4:** De novo* a*ssembly metrics for the four isolates with supplementary long-read sequencing

Isolate code	Species identification	No'contigs	Chromosome, complete	Size of plasmid with carbapenemase ARG(s)	ARGs on plasmid	Plasmid type/BLAST similarity to
PW0012.1	*Acinetobacter baumannii*	7	3,820,549 bp, Y	309,568 bp, Y	*ARR-3*, *sul1*, *bla*_NDM-1_, *aph(3′)-Via*, *bla*_CARB-16_, *aac(3)-Iid*, *aph(3″)-Ib*,* aph(6)-Id*, *mph(E)*, *msr(E)*, *tet*(X3), *floR*, *sul2*	NZ_CP042365.1
PW0012.2	*Klebsiella pneumoniae*	8	5,300,304 bp, Y	273,549 bp, Y	*bla*_NDM-1_, *bla*_OXA-48_, *aph(3')-VI*, *mph(E)*, *msr(E)*, *armA*, *sul1*, *dfrA12*	IncHI1B/IncFIB, NZ_CP050156.1
PW0068	*Acinetobacter* sp. (30% unclassified reads)	12	2,799,419 bp, Y	53,570 bp, Y	*tet*(39), *mph(E)*, *msr(E)*, *bla*_NDM-1_, *aph(3')-VI*, *bla*_OXA-58_, *sul1*	NZ_LC591943.1
OG0025	*Acinetobacter baumannii*	26	3,657,799 bp, Y	268,855 bp, N	*bla*_NDM-1_, *sul2*, *bla*_OXA-23_, *msr(E)*, *mph*(E), *tet*(39), *aac(3)-Iid*	NZ_MK134375.1

The number of contigs, the size of bacterial chromosome and plasmid with antibiotic resistance genes (ARG) of interest are shown (Y, indicates the chromosome/plasmid was circularised/complete; N, indicates the contig is linear). All ARG on the target plasmid are listed and the plasmid inc type is shown were applicable. The most similar plasmid as per mash dist analysis in PLSDB is listed

All 25 Acinetobacter isolates were *bla*_NDM-1_ positive and the hybrid assembly of three different *Acinetobacter* isolates reveals different size plasmids circulating in the hospital environment all containing *bla*_NDM_ (Table 4). The *bla*_NDM-1_ gene in a 309,568 bp plasmid in an *A. baumannii*, ST132 isolate (PW0012-1) was carried by the transposon Tn125. The isolate identified to *Acinetobacter* sp. by WGS carried a 53,570 bp plasmid with *bla*_NDM-1_ and *bla*_OXA-58_, whereas a 268,855 bp plasmid carried by ST109 *A. baumannii* co-harboured *bla*_NDM-1_ and *bla*_OXA-23_ (Table 4). Regardless of the plasmid genomic context, *ble*_MBL_ (a gene conferring resistance to bleomycin and bleomycin-like antibiotics) was identified downstream of all four isolates.

There were 14 different *bla*_OXA_ variants (both ESBL and carbapenemase variants) detected with n = 24/25 (96%) of *Acinetobacter* genomes containing carbapenem-hydrolysing oxacillinase (CHO) ARG including *bla*_OXA-23_, *bla*_OXA-58_, and *bla*_OXA-67_ (*bla*_OXA-51_-like), although *bla*_OXA-67_ were only detected in the ST109 *A. baumannii* cluster (Table 3, Fig. 3). Six *Acinetobacter* from six different species carried *bla*_OXA-58_ however *bla*_OXA-58_ was not detected in *A. baumannii* isolates whereas *bla*_ADC_ was detected in all *A. baumannii* isolates only. Whilst there is no *Acinetobacter* EUCAST breakpoint or ECOFF value for cefotaxime, the MIC values obtained were very high, ranging between 128 and  > 256 mg/L (Table 3).

Resistance to gentamicin was high (n = 25/27, 93% [n = 27 for MIC, n = 28 with WGS/ARG data) with 56 aminoglycoside ARG from 13 variants found (Table 3, Fig. 3). The most frequent were *aac*(3)-IId (n = 19/28, 68%), *aph*(6′)-Id (n = 8/28, 29%) and *aph*(3′)-Id (n = 7/28, 25%). On the other hand, resistance to colistin was low overall (n = 2/27, 7%), colistin resistance was observed within the *K. pneumoniae* isolate (PW0012.2; MIC 32 mg/L) and an *Acinetobacter junii* isolate (PW0056; 8 mg/L). Only one isolate (n = 1/27, 4%) had a tigecycline MIC of 8 mg/L, an *A. baumannii* harbouring a *tet*(X3) gene (PW0012.1), however (n = 9/26, 35%) *Acinetobacter* isolates (inclusive of PW0012.1) had resistant MICs ranging between 1 and 8 mg/L. Furthermore, we detected *tet*(X3) in an additional isolate, identified as *A*. *johnsonii*, although this was sensitive to tigecycline (MIC 0.5 mg/L). The two *Acinetobacter* carried *tet*(X3) at 100% coverage and 100% identity to the reference, a mobile resistance gene that confers resistance to tigecycline. The *A. baumannii* isolate with *tet*(X3) (PW-0012.1) was typed as ST132 (ST1181 Oxford scheme). Complementary long read sequencing and annotation of the complete plasmid with *tet*(X3) reveals the *tet*(X3) gene is carried on the same plasmid as *bla*_NDM-1_. Only short read WGS was available for the *Acinetobacter johnsonii* isolate positive for *tet*(X3), genetic context could not be extrapolated as the contig containing the *tet*(X3) was 4304 bp.

In terms of additional ARGs detected in the WGS data, trimethoprim (*dfrA1*, *dfrA12*, *dfrA14*, and *dfrA20*) ARGs were detected in four species including *A. baumannii, A. indicus* and *K. pneumoniae* whereas fluoroquinolone ARG (*OqxA/B*, *qnrB* and *qnrVC1*) were detected in two isolates, *K. pneumoniae* and one *P. stutzeri*. Macrolide ARG (*mph(E)* and *msr(E)*) were detected in (n = 26/28, 93%) and sulphonamide ARG (*sul1* and *sul2*) were detected in all isolates except the *P. stutzeri* isolates.

### Antimicrobial resistance data for patient isolates collected locally at CCTH

Ninety-five patient isolates were collected between 1st January to 30th June 2021 (pre- and post-swab collection) and these consisted of 48. *Escherichia coli*, 26 *Klebsiella* spp., 1 K*. oxytoca*, 2 K*. pneumoniae*, 5 *Enterobacter* spp., 12 *Pseudomonas spp.* and 1 *P. aeruginosa* isolates. MICs were determined against ampicillin-sulbactam, carbapenems (ceftriaxone or cefotaxime), and piperacillin/tazobactam. All isolates tested were resistant to both ceftriaxone and cefotaxime, with the exception of 7/45 *E. coli* isolates examined, indicative of prevalent ESBLs and carbapenemases consistent with our environmental swab findings. A smaller sub-set (N = 27, due to restricted availability of antimicrobial disks) was examined for susceptibility to β-lactam + β-lactamase inhibitor combinations, and also showed 100% resistance except for 1/12 of the *E. coli* isolates. However, no data was recorded for *Acinetobacter* spp. during this period because the local microbiology laboratory is unable to identify this species through the limited available biochemical tests available locally. *Acinetobacter* spp. could have been recorded as *Enterobacteriaceae*, *Enterobacter* spp. or *Citrobacter* spp. based on the local SOP as *Acinetobacter* spp. was not one of the potential determinants for the biochemical outcome flowchart. Therefore, no AST values can be specifically assigned to *Acinetobacter* for comparison.

## Discussion

This article reports data from a pilot study evidencing hospital surface colonisation with MDR GNB across five different wards; AE, FMW NICU, OG, and PW. Importantly, local scientists and members of the hospital staff collected the clinical environmental swabs without notice, limiting the use of disinfectant immediately before sampling or other behavioural bias among staff responsible for disinfection (e.g., the Hawthorne effect). PCR-confirmed ESBL and carbapenemase-producing isolates were recovered from all wards except FMW where all screened isolates produced negative results for AMR genes of interest despite high growth rates of 67% VE and 47% plates from swabs taken from the ward’s sampled environments during screening (Table 1, Fig. 1). Although the FMW ward is proximal to the other sampled wards the difference in terms of ARG detection indicates variation among environmental contaminating bacteria within a single institution. One of the most striking findings of this study was the high growth of bacteria in the presence of cefotaxime and meropenem and the diversity of *Acinetobacter* species (Fig. 3) harbouring multiple carbapenemase antibiotic resistance genes, noticeably *bla*_NDM-1_, *bla*_OXA-23_, *bla*_OXA-58_ and *bla*_OXA-67_. CRAB have often been linked to hospital-acquired infections (HAI) [9, 20, 65, 66] and have regularly been detected colonising abiotic hospital surfaces [67]. We also detected *A. nosocomialis* within the PW on thermometers and computer keyboards suggesting multiple carbapenem-resistant *Acinetobacter* species (CRAS) are present with potential to transmit within the wards. Of the 10 different *Acinetobacter* species identified in this study in total, eight harboured *bla*_NDM-1_ in addition to CHO. Similarly, Bhatta et al*.* performed a study in a tertiary care hospital in Nepal whereby 232 samples were collected from various sites and *Acinetobacter* species were the most prevalent GNB [68], and Aliramezani et al. detected clonal expansion of CRAB in hospital environments in Iran [69]. There are limited published data available from Africa, however high emerging prevalence of carbapenemase *A. baumannii* has been documented in wound infections in Ghana [32, 33].

Collectively CRAS were recovered from a wide variety of high-touch hospital surfaces including wall sockets, cots, mattresses, sheets, pillows, computer keyboards, medical equipment, bed handles and tap handles. This finding is in accordance with previous studies suggesting *Acinetobacter* successfully colonise abiotic surfaces [70]. The genetic relationship between the cluster of ST109 *bla*_NDM-1_ positive *A. baumannii* isolates identified in OG was investigated with a mapping-based SNP analysis using a local complete reference genome to improve coverage and SNP calling across the whole genome [71]. All ST109 *A. baumannii* were within 6 pairwise SNPs, with four identical isolates (0 SNPs), suggesting the spread of this strain throughout the OG ward. Scanning the PubMLST database [72] revealed that only 5/6640 (on 23 July 2021) isolates submitted were ST109 (Pasteur scheme) suggesting this is a relatively uncommon ST globally. *Acinetobacter baumannii* can persist in the healthcare environment by tolerating desiccation and producing biofilms on abiotic surfaces [70]. CRAB isolates recovered in this study were screened for biofilm-associated VF and detection of *bap* genes indicates biofilm production was possible. Future studies combining biofilm production assays and WGS could be beneficial to determine whether specific STs have the potential to persist longer in the environment. Studies combining antibiotic susceptibility testing, PFGE and biofilm assays have shown distinct clusters of CRAB persisting in the environment, including in high-risk wards like NICU [69]. During this study we identified three carbapenem resistant isolates, including *Acinetobacter* and *Pseudomonas* from cots, a medical trolly and bed wheels. The detection of a smaller cluster (three) of *A. noscomialis* isolates from a different ward, PW indicates multiple introductions of MDR Acinetobacter, and all these isolates originated from swabs from pieces of equipment regularly used by hand (Table 3). Unfortunately, the limited diagnostic capacity for the accurate speciation of certain isolates which include *Acinetobacter baumannii*, *Pseudomonas* spp. (which do not produce pigments), *Klebsiella* spp. (aside from that of *K. pneumoniae* and *Enterobacter* spp.) in the CCTH microbiology lab would likely result in misidentification. Four biochemical tests namely; indole, urease, citrate, and triple sugar iron test are utilised in this regard. This limited panel could lead to the misidentification of species especially within gram-negative non-fermenters due to similarities in the test outcomes especially for *Pseudomonas* spp. and *Acinetobacter* spp. Furthermore, the lack of PCR and visualisation equipment currently make it impossible to screen for ARGs. Rectifying these short-falls, especially in light of the findings for this study, are currently being addressed.

In 2019 He et al. first reported and characterised two mobile resistance genes named *tet*(X3) and *tet*(X4) which were isolated from (food-producing) animals in China [73]. When they performed genomic data mining, they found the presence of both *tet*(X3) in *Acinetobacter* and *tet*(X4) in Enterobacterales isolated from several countries including Cote d’Ivoire predating 2014 suggesting the dissemination into human pathogens of these genes. Here we report two *Acinetobacter* isolates carrying *tet*(X3) in addition to *bla*_NDM_. We often cultured multiple bacterial isolates from the same sample, containing a mixture of ESBL and/or carbapenemase genes (Fig. 2); most notably in this study a particular sample PW0012, a swab of the top surface and drawer handles of a bedside cabinet in the east wing of PW. It is concerning that bacterial isolates from this sample alone were resistant to aminoglycosides, cephalosporins, β-lactams, carbapenems, tigecycline and colistin. To the best of our knowledge this is the first report of multidrug-resistant *Acinetobacter* also harbouring *tet*(X3) in Ghana.

CCTH in Ghana has around 16 inpatient wards and this study collected 231 swabs across five wards over one month from March 2021. Surveillance across all wards is vital to determine which wards are at higher risk of greater MDR colonisation rates and such data is invaluable to focus on interventional infection control policies. Currently, the hospital performs infection prevention and control practices according to the national policy’s technical guidelines encompassing hand hygiene, environmental management and controls, handling linen, and environmental and engineering considerations which state the appropriate disinfectants/antiseptic agents to use and the procedures for using them. Some wards uphold more rigorous cleaning schedules and implement additional cleaning requirements, depending on the presence of certain equipment and other demands. However, there is limited infection control surveillance in the laboratory and inadequate monitoring and evaluation mechanisms in place to stringently enforce these IPC policies and practices. Data generated from such a study places emphasis on the need to implement increased measures to limit the spread of AMR. Limitations of this study include a short sampling period, small sample sizes for both swab sites and wards, and selective WGS, however the detection of clusters of closely related CRAS, mobile tigecycline resistance genes, ESBL and carbapenemase gene within *K. pneumoniae, E. cloacae,* and *E. coli* warrants larger and continued surveillance to reduce possible transmission events between hospital surfaces and patients. It would be beneficial for further studies to include sampling of health care workers’ hands, especially those working across multiple wards in a given time period, and also to link to potential HAI and AMR cases within the hospital. Future surveillance studies adopting a ‘One-Health’ approach including local community AMR surveillance are essential to provide context and linkage across all sectors [74] to provide data to inform intervention and policy change.

## Conclusion

A high level of AMR-containing species and contaminated surfaces were detected within the hospital environment, noticeably in the OG and PW wards. A diversity of resistance mechanisms and species were detected within the hospital environment, including isolates with multi-drug resistance and evidence multiple of localised clonal transmission. Many of the surfaces swabbed harboured bacteria of multiple species carrying different ARG conferring resistance to several classes of antibiotics. This indicates surface colonisation could be acting as a potential reservoir for the transmission of MDR pathogens within the hospital environment. Noting high rates of healthcare-associated infections in sub-Saharan Africa and the role hospital environments play in these as evidenced in studies globally, environmental surveillance is an invaluable tool in reducing its incidence thus reducing AMR rates in the region. In Ghana, the National Action Plan for Antimicrobial Use and Resistance policy in strategic objective 7.1.1.1 focuses on the implementation of the National IPC policy. This study builds the needed foundation for the establishment of routine environmental surveillance as a key indicator for evaluating IPC practices within healthcare institutions in the country.

## Supplementary Information

**Additional file 1**. Additional data for antimicrobial resistance testing of patient isolates of Gram-negative bacteria isolated before, during and after the environmental swabs were collected, collated by corresponding wards sampled.

## Data Availability

All fastq reads (Illumina) were submitted to the ENA repository under the project accession PRJEB46496. Hybrid genomes were upload to the NCBI under the project accession PRJNA750808.
