# Supplementary material for: Environmental surveillance of ESBL and carbapenemase-producing gram-negative bacteria in a Ghanaian Tertiary Hospital
Source: Antimicrob Resist Infect Control. 2022 Mar 16;11:49. doi: 10.1186/s13756-022-01090-2 (PMC8925048; doi:10.1186/s13756-022-01090-2)
Supplement: Supplementary file 1 — Additional file 1. Additional data for antimicrobial resistance testing of patient isolates of Gram-negative bacteria isolated before, during and after the environmental swabs were collected, collated by corresponding wards sampled. [file 13756_2022_1090_MOESM1_ESM.docx]

**Additional file**

Additional file 1: Table S1. The PCR primer sequences, amplicon size, reference and control isolates used.

| **Primer pairs** | **Target** | **Sequence (5’-3’)** | **Amplicon size (bp)** | **Reference** | **Control Strain** |
| --- | --- | --- | --- | --- | --- |
| CTXM15- F | *bla*CTX-M-15 | ATGCGCAAACGGCGGACGTA | ~600 | Walsh group (Carvalho MJ) | *Escherichia coli* NCTC 13353 |
| CTXM15- R |  | CCCGTTGGCTGTCGCCCAAT |  |  |  |
| NDM-M-F | *bla*NDM | AGCTGAGCACCGCATT | 648 | Walsh group (Hassan B) | Escherichia coli (in-house control strain) |
| NDM-M-R |  | CTCAGTGTCGGCATCAC |  |  |  |
| KPC-M-F | *bla*KPC | TAGTTCTGCTGTCTTGTCTC | 333 | Walsh group (Hassan B) | *Klebsiella* sp. K10 (in-house control strain) |
| KPC-M-R |  | CCGTCATGCCTGTTGTC |  |  |  |
| OXA-48-M-F | *bla*OXA-48 (and OXA-48-like genes: *bla*OXA-162, -163, -181, and possibly -204 & -232) | GGCGTAGTTGTGCTCTG | 155 | Walsh group (Hassan B) | *K. pneumoniae* NCTC 13442 |
| OXA-48-M-R |  | AAGACTTGGTGTTCATCCTT |  |  |  |
| TEM-F | *bla*TEM variants including TEM-1 and TEM-2 | CATTTCCGTGTCGCCCTTATTC | 800 | Dallenne et al., 2010 | *Klebsiella* sp. (in-house control strain) |
| TEM-R |  | CGTTCATCCATAGTTGCCTGAC |  |  |  |
| SHV-F | *bla*SHV variants including SHV-1 | AGCCGCTTGAGCAAATTAAAC | 713 | Dallenne et al., 2010 | *Klebsiella* sp. (in-house control strain) |
| SHV-R |  | ATCCCGCAGATAAATCACCAC |  |  |  |
| OXA-1-F | *bla*OXA-1, OXA-4 and OXA-30 | GGCACCAGATTCAACTTTCAAG | 564 | Dallenne et al., 2010 | *Klebsiella* sp. (in-house control strain) |
| OXA-1-R |  | GACCCCAAGTTTCCTGTAAGTG |  |  |  |

Additional file 1: Table S2. The PCR thermocycling conditions

|  | CTX-M-15 | | (NDM/OXA/KPC) | | TEM/SHV/OXA-1 | |
| --- | --- | --- | --- | --- | --- | --- |
| Cycles | Temperature °C | Time (Minutes) | Temperature °C | Time (Minutes) | Temperature °C | Time (Minutes) |
| 1x | 95 | 05:00 | 95 | 05:00 | 94 | 10:00 |
| 30x | 94 | 00:30 | 95 | 00:30 | 94 | 00:40 |
|  | 52 | 01:00 | 61 | 00:30 | 60 | 00:40 |
|  | 72 | 01:00 | 72 | 00:30 | 72 | 01:00 |
| 1x | 72 | 10:00 | 72 | 10:00 | 72 | 07:00 |

Additional file 1: Table S3. SOP for biochemical identification of gram-negative bacteria in the CCTH microbiology lab.

|  | Indole | citrate | urea | glucose | lactose | Hydrogen sulphide | Gas | Oxidase | Motility |
| --- | --- | --- | --- | --- | --- | --- | --- | --- | --- |
| *Escherichia coli* | + | - | - | + | + | - | - | - | - |
| *Enterobacter spp.* | - | +/- | - | + | + | - | + | - | + |
| *Klebsiella spp.* | - | + | + | + | + | - | + | - | - |
| *Pseudomonas spp.* | - | + | +/- | +/- | - | - | - | + | + |
| ***Shigella spp.*** | **+** | **-** | **-** | **+** | **-** | **-** | **-** | **-** | **-** |
| ***Salmonella typhi*** | **-** | **-** | **-** | **+** | **-** | **-** | **-** | **-** | **-** |
| ***Salmonella paratyphi*** | **-** | **-** | **-** | **+** | **-** | **+/-** | **-** | **-** | **+** |
| ***Salmonella spp. (others)*** | **-** | **+/-** | **-** | **+** | **+** | **+/-** | **+** | **-** | **+/-** |
| ***Citrobacter spp.*** | **+/-** | **+/-** | **+/-** | **+** | **+** | **+/-** | **+** | **-** | **+** |
| ***Serratia marcescens*** | **-** | **+** | **+/-** | **+** | **+/-** | **-** | **+/-** | **-** | **+** |
| ***Proteus vulgaris*** | **+** | **+/-** | **+** | **+** | **-** | **+** | **+/-** | **-** | **+** |
| ***Proteus mirabilis*** | **-** | **+/-** | **+** | **+** | **-** | **+** | **+/-** | **-** | **+** |
| ***Morganella morganii*** | **+** | **-** | **+** | **+** | **-** | **-** | **+** | **-** | **+/-** |
| ***Providencia spp.*** | **+** | **+** | **+/-** | **+** | **-** | **-** | **+/-** | **-** | **+** |

**For speciation of *Klebsiella spp*. and *Pseudomonas spp.***

1. *K. oxytoca* – acidic TSI slant, citrate positive, H_2_S negative, indole positive
2. *K. pneumoniae* - acidic TSI slant, citrate positive, H_2_S negative, indole negative, positive butt Lysine decarboxylase test (test not routinely performed in the laboratory)
3. *Pseudomonas aeruginosa* – same as *Pseudomonas* spp. with the production of pigment

Additional file 1: Table S4. The number of swabs collected per sample source (area swabbed), the number and percentage of growth on chromogenic agar containing vancomycin and cefotaxime (VC) and agar containing vancomycin and meropenem (VM).

| **Area swabbed** | **Number of swabs** | **Number growth on VC** | **Number growth on VM** | **% growth VC** | **% growth VM** |
| --- | --- | --- | --- | --- | --- |
| Bed cart | 13 | 13 | 11 | 100 | 85 |
| Bed handle | 8 | 6 | 5 | 75 | 63 |
| Bed handle (cot) | 4 | 3 | 3 | 75 | 75 |
| Bed sheet | 6 | 6 | 3 | 100 | 50 |
| Bed tray | 9 | 9 | 9 | 100 | 100 |
| Bed wheels | 10 | 9 | 8 | 90 | 80 |
| Bedpan | 3 | 3 | 2 | 100 | 67 |
| Bedside basket | 2 | 1 | 0 | 50 | 0 |
| Bedside cabinet top and drawers | 8 | 8 | 8 | 100 | 100 |
| Computer keyboard | 13 | 12 | 9 | 92 | 69 |
| Desk surface | 13 | 11 | 9 | 85 | 69 |
| Incubator | 1 | 1 | 0 | 100 | 0 |
| IV stand | 13 | 9 | 6 | 69 | 46 |
| Light switch | 13 | 6 | 3 | 46 | 23 |
| mattress | 7 | 6 | 5 | 86 | 71 |
| Medicine trolley | 3 | 1 | 1 | 33 | 33 |
| Pillow | 7 | 7 | 7 | 100 | 100 |
| Procedure table surface | 2 | 2 | 2 | 100 | 100 |
| Pulse oximeter | 5 | 2 | 2 | 40 | 40 |
| Sphygmomanometer | 5 | 5 | 4 | 100 | 80 |
| Stethoscope | 10 | 4 | 2 | 40 | 20 |
| Tap handle | 17 | 14 | 12 | 82 | 71 |
| Thermometer | 10 | 9 | 6 | 90 | 60 |
| Waiting area chair handle | 12 | 10 | 7 | 83 | 58 |
| Wall socket switch | 12 | 7 | 5 | 58 | 42 |
| Washroom door handle | 13 | 10 | 8 | 77 | 62 |
| Weighing scale | 1 | 0 | 1 | 0 | 100 |
| Window levers | 11 | 6 | 3 | 55 | 27 |
| **Total** | **231** | **180** | **141** | **78%** | **61%** |
